# Continuity in general practice as predictor of mortality, acute hospitalisation, and use of out-of-hours care: a registry-based observational study in Norway

**DOI:** 10.3399/BJGP.2021.0340

**Published:** 2021-10-05

**Authors:** Hogne Sandvik, Øystein Hetlevik, Jesper Blinkenberg, Steinar Hunskaar

**Affiliations:** National Centre for Emergency Primary Health Care, NORCE Norwegian Research Centre, Bergen.; Department of Global Public Health and Primary Care, University of Bergen, Bergen.; National Centre for Emergency Primary Health Care, NORCE Norwegian Research Centre, Bergen; Department of Global Public Health and Primary Care, University of Bergen, Bergen.; NORCE Norwegian Research Centre, Bergen; Department of Global Public Health and Primary Care, University of Bergen, Bergen.

**Keywords:** continuity of patient care, emergency medical services, family practice, general practice, hospitalisation, mortality, Norway

## Abstract

**Background:**

Continuity, usually considered a quality aspect of primary care, is under pressure in Norway, and elsewhere.

**Aim:**

To analyse the association between longitudinal continuity with a named regular general practitioner (RGP) and use of out-of-hours (OOH) services, acute hospital admission, and mortality.

**Design and setting:**

Registry-based observational study in Norway covering 4 552 978 Norwegians listed with their RGPs.

**Method:**

Duration of RGP–patient relationship was used as explanatory variable for the use of OOH services, acute hospital admission, and mortality in 2018. Several patient-related and RGP-related covariates were included in the analyses by individual linking to high-quality national registries. Duration of RGP–patient relationship was categorised as 1, 2–3, 4–5, 6–10, 11–15, or >15 years. Results are given as adjusted odds ratio (OR) with 95% confidence intervals (CI) resulting from multilevel logistic regression analyses.

**Results:**

Compared with a 1-year RGP–patient relationship, the OR for use of OOH services decreased gradually from 0.87 (95% CI = 0.86 to 0.88) after 2–3 years’ duration to 0.70 (95% CI = 0.69 to 0.71) after >15 years. OR for acute hospital admission decreased gradually from 0.88 (95% CI = 0.86 to 0.90) after 2–3 years’ duration to 0.72 (95% CI = 0.70 to 0.73) after >15 years. OR for dying decreased gradually from 0.92 (95% CI = 0.86 to 0.98) after 2–3 years’ duration, to 0.75 (95% CI = 0.70 to 0.80) after an RGP–patient relationship of >15 years.

**Conclusion:**

Length of RGP–patient relationship is significantly associated with lower use of OOH services, fewer acute hospital admissions, and lower mortality. The presence of a dose–response relationship between continuity and these outcomes indicates that the associations are causal.

## INTRODUCTION

Continuity is a core value of primary care. McWhinney described continuity as an implicit contract between a patient and a GP, who then takes personal responsibility for the patient’s medical needs.^1^^,^^2^ Continuity is not limited by the type of disease and bridges episodes of various illnesses. Greater continuity with a primary care physician has been shown to be associated with lower mortality rates,^3^ fewer hospital admissions,^4^^,^^5^ less use of emergency departments,^6^ and fewer referrals for specialist health care.^7^^,^^8^ Nevertheless, continuity has been declining in recent years.^9^

There is no uniform agreement about how continuity should be defined, but three aspects are usually described: informational, longitudinal, and interpersonal.^10^ Informational continuity means that the doctor has adequate access to all relevant information about the patient. Longitudinal continuity means that it transcends multiple episodes of illness, and interpersonal refers to a trustful relationship between patient and physician. Various methods have been used for measuring continuity. Most of them are based on visit patterns with different providers over time.^10^^,^^11^ An example is the Usual Provider of Care (UPC) index, which calculates the percentage of all contacts that is with the most frequent provider.^12^ Most of these studies have been conducted with limited patient samples and rather short observation periods. There is scarce literature on studies with large- or full-scale populations, long follow-up, and hard endpoints.

In a limited number of countries, such as the UK, the Netherlands, Denmark, or Norway, most inhabitants are listed with a general practice or a named regular general practitioner (RGP) who is responsible for taking care of their medical needs. Such RGP schemes are usually established not only to increase continuity of care as an assumed aspect of quality, but also to prevent unnecessary spending by introducing the RGP as a gatekeeper. It should be noted, however, that patients also value such personal relationships with their RGP.^13^

The aim of the present study, based on Norwegian registry data, was to analyse, on a national level, the effects of longitudinal RGP continuity associated with use of out-of-hours (OOH) services, acute hospital admissions, and mortality.

## METHOD

### The Norwegian RGP Scheme

In Norway, the state is responsible for hospitals, while the primary healthcare system is the responsibility of the municipalities. In 2018 there were >400 municipalities in Norway.

**Table:** How this fits in

Continuity of care with a GP is generally regarded as an aspect of quality. It is usually measured by visit patterns with different providers over time and is associated with lower mortality rates, fewer hospital admissions, and less use of emergency departments. This nationwide study of the Norwegian population shows that longitudinal continuity with a named regular GP is significantly associated with the need for out-of-hours services, acute hospital admissions, and mortality in a dose-dependent way. When longitudinal continuity exceeds 15 years, the probability of these occurrences is reduced by 25–30%.

In 2001 a reform called the RGP Scheme, a contractual system based on listing and capitation, was introduced.^14^ All inhabitants were invited to choose their own RGP. Those who did not express any preference were assigned to an RGP with available capacity. More than half of the patients stated that it was most important to keep a GP they already knew. This was especially important for older people with poor health.

As it transpired, 87.3% were assigned to their first choice and the mean list size was 1175.^14^ Only 0.2% of the inhabitants decided not to participate in the RGP Scheme.^15^ RGPs are gatekeepers; patients cannot self-refer themselves to hospital, an outpatient clinic, or an emergency department. Patients can change RGP a maximum of twice a year. When this happens, the medical record will usually be transferred to the new RGP. People residing in nursing homes are still formally registered with their last RGP, even if the medical responsibility is transferred to the attending physician at the nursing home.

Most RGPs work in small group practices of 3–6 doctors. However, RGPs also do public medical work in nursing homes, prisons, schools, and maternal and child health centres, and are therefore usually present in their practices 3–4 days a week. Colleagues usually help each other, seeing patients who cannot or will not wait for their own RGP to be present. Many practices also have interns. Informational continuity is secured since group practices have common electronic patient records.

OOH services are also the responsibility of municipalities. Some municipalities have their own OOH service, while others cooperate. In 2018 there were 177 different OOH services in Norway: 75 municipal and 102 intermunicipal cooperatives.^16^ OOH services are mainly staffed by RGPs, but other physicians may also participate.

Both RGP practice and OOH services are based on fee for service. As for out-of-pocket expenses, children younger than 16 years pay nothing. Others usually pay €20–30 (£17–26), depending on the service offered. In addition, doctors always send electronic compensation claims to the Norwegian Health Economics Administration (HELFO). The third component of RGPs’ income is capitation paid by the municipality, about €50–60 (£43–52) yearly per inhabitant on their list.

### Study population and data sources

All Norwegian citizens are given a unique personal identification number (ID-number) at birth. This number is used in various official records and allows for linking such records on an individual level. Foreigners moving to Norway and wishing to stay for more than 6 months are also given an ID-number. This study’s sample was created by linking information from four nationwide registries by ID-number, allowing several possible confounders to be included in addition to the main variables. The present study is based on data from 2015–2018. The following data sources were used:

- Statistics Norway (SSB);
- Control and Payment of Reimbursement to Health Service Providers (KUHR);
- the Norwegian Patient Registry (NPR); and
- the RGP registry.

SSB provided demographic data about all inhabitants, such as sex, age, education, centrality, ethnicity, and deaths during 2018. In 2018 the population of Norway was approximately 5.3 million. Highest fulfilled education was categorised as none or elementary, upper secondary, or higher education. Centrality describes a municipality’s geographical location in relation to a centre where there are important functions (central functions). It is categorised from 1 (most urban) to 6 (most rural). Ethnicity was categorised in three groups (country of birth):

- Norway;
- an immigrant from the EU, the US, Canada, New Zealand, or Australia; and
- an immigrant from the rest of Europe, Asia, Africa, or Latin America.

The KUHR database is maintained by HELFO and has complete records of all patient contacts with RGPs and OOH services in Norway. For this study it was recorded if the person had contacted the OOH services during 2018, defined as having ≥1 consultation at the OOH clinic or a home visit.

The NPR database contains information about all patient contacts with specialist health care, including hospital admissions. For this study it was recorded if the person had been acutely admitted to somatic hospital during 2018. Birth-related admissions were excluded (ICD-10 codes Z37 and Z38).

Morbidity was defined by the Royal College of Surgeons Charlson Score, which is based on 14 groups of ICD-10 codes used in specialist and hospital care (Table 1).^17^ Any use of the relevant ICD-10 codes during the three preceding years (2015–2017), be it outpatient or inpatient, was recorded (also NPR database).

**Table 1.** Morbidity was defined by the Royal College of Surgeons Charlson Score, which is based on 14 groups of ICD-10 codes used in specialist and hospital care^17^

**Condition**	**ICD-10 codes**
Myocardial infarction	I22–23, I252
Congestive heart failure	I11, I13, I255, I42–43, I50, I517
Peripheral vascular disease	I70–73, I770–I771, K551, K558–559, R02, Z958–959
Cerebrovascular disease	G45–46, I60–69
Dementia	A810, F00–03, F051, G30–31
Chronic pulmonary disease	I26–27, J40–47, J60–67, J684, J701, J703
Rheumatic disease	M05–06, M09, M120, M315, M32–36
Liver disease	B18, I85, I864, I982, K70-71, K721, K729, K76, R162, Z944
Diabetes mellitus	E10–14
Hemiplegia/paraplegia	G114, G81–83
Renal disease	I12-13, N01, N03, N05, N07–08, N171–172, N18–19, N25, Z49, Z940, Z992
Malignancy	C00–26, C30–34, C37–41, C43, C45–58, C60–76, C80–85, C88, C90–97
Metastatic tumours	C77–79
HIV/AIDS	B20–24

The RGP registry contains information about all RGPs and their listed patients. For this study the RGP’s sex, age, and whether they are an approved general practice specialist were recorded. List size (the number of persons listed with each RGP) and the number of vacant list places for new patients were also recorded. This is the difference between the maximum number of persons the RGP will accept, and the actual number of persons listed. Finally, how many years the RGP–patient relationship had lasted was recorded: 1 year, 2–3 years, 4–5 years, 6–10 years, 11–15 years, or >15 years. These RGP data were recorded at the start of 2018. There were 5 301 036 persons who were assigned to an RGP at this time, but 748 058 who changed RGP during 2018 were excluded, leaving 4 552 978 for analysis.

### Statistics

Three multiple logistic regression analyses were performed, with the duration of the RGP–patient relationship as the main explanatory variable. The three dependent variables were: use of OOH services (at least one consultation or home visit), hospital admission (at least one acute admission), and death (all in 2018).

The following patient variables were included as covariates: sex, age (continuous), educational level, country of birth, Charlson score (continuous), centrality, and frequency of RGP visits (continuous). The following RGP variables were used: sex, age (continuous), general practice specialist or not, list size (continuous), and vacant list capacity (continuous). Adjustments were first performed for patient variables only; thereafter, RGP variables were also added. Because of the clustered nature of the material (patients clustered by individual RGPs) the data were analysed by multilevel logistic regression.^18^ Results are presented as odds ratios (OR) with 95% confidence intervals (CI). The analyses were carried out by using Stata (version 16).

## RESULTS

**Figure 1.**
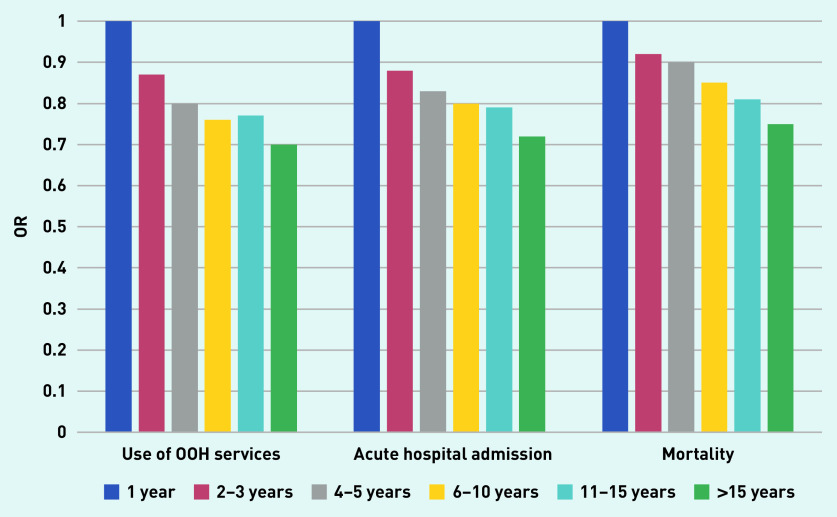
*Associations between continuity measured as years with the same RGP and odds for use of OOH services, acute hospital admissions, and mortality during 2018.* *OOH = out-of-hours. OR = odds ratio.*

A description of the material according to duration of RGP–patient relationship is given in Table 2. Mean age of the 4708 included RGPs was 47.7 years, 57.8% were male, and 61.3% were general practice specialists. Mean list size was 1113 and mean vacant list capacity was 49.4. Table 3 shows that there was a consistent and significant trend towards less use of OOH services with increasing duration of the RGP–patient relationship. Compared with a 1-year RGP–patient relationship, the OR for use of OOH services decreased gradually from 0.87 (95% CI = 0.86 to 0.88) after 2–3 years’ duration to 0.70 (95% CI = 0.69 to 0.71) after >15 years (Figure 1). Table 4 shows a similar consistent trend for acute hospital admissions. OR for acute hospital admissions decreased gradually from 0.88 (95% CI = 0.86 to 0.90) after 2–3 years’ duration to 0.72 (95% CI = 0.70 to 0.73) after >15 years.

**Table 2.** Description of patients and their regular GPs (RGPs) by duration of RGP–patient relationship^a^

	**Duration of RGP–patient relationship**						

	**1 year (*n*= 609 577)**	**2–3 years (*n*= 868 490)**	**4–5 years (*n*= 647 761)**	**6–10 years (*n* = 955 974)**	**11–15 years (*n* = 667 154)**	>**15 years (*n* = 804 022)**	**Total (*n* = 4 552 978)**
**Patient variables**							
Male patients, (*n*) %	(297 150) 48.7	(434 958) 50.1	(328 433) 50.7	(489 029) 51.2	(348 618) 52.3	(406 857) 50.6	(2 305 045) 50.6
Mean age patients, years	35.1	36.6	38.2	39.8	38.9	56.3	41.1
Higher education, (*n*) %^b^	(153 875) 36.2	(221 752) 36.0	(167 389) 35.4	(249 637) 34.6	(164 334) 29.3	(248 200) 31.1	(1 205 187) 33.5
Norwegian born, (*n*) %	(478 151) 78.4	(682 010) 78.5	(528 164) 81.5	(805 794) 84.3	(608 008) 91.1	(752 918) 93.6	(3 855 045) 84.7
Mean Charlson score (0 = min, 14 = max)	0.15	0.16	0.17	0.17	0.15	0.26	0.18
Mean centrality (1 = rural, 6 = urban)	2.9	2.9	2.7	2.7	2.7	2.7	2.8
Mean number of consultations per year	2.6	2.7	2.8	2.7	2.5	3.1	2.7

**RGP variables**							
Male RGP, (*n*) %	(327 046) 53.7	(481 071) 55.4	(361 730) 55.8	(570 307) 59.7	(419 509) 62.8	(578 438) 71.9	(2 738 101) 60.1
Mean age RGP years	42.3	43.4	45.0	48.2	53.5	59.4	48.8
GP specialist, (*n*) %	(250 787) 41.1	(387 863) 44.6	(373 786) 57.7	(736 221) 77.2	(573 695) 85.9	(706 800) 87.9	(3 0291 52) 66.4
Mean list size (number of listed persons)	1140	1180	1215	1263	1306	1340	1244
Mean vacant list capacity (number of places)	70.1	44.9	26.9	19.8	12.3	8.5	29.1

**Patient outcome variables**							
Use of OOH services, (*n*) %	(121 372) 19.9	(157 056) 18.1	(107 172) 16.5	(147 236) 15.4	(104 611) 15.7	(122 126) 15.2	(759 573) 16.7
Acute hospital admission, (*n*) %	(39 014) 6.4	(52 716) 6.1	(38 026) 5.9	(56 180) 5.9	(37 553) 5.6	(65 822) 8.2	(2 893 11) 6.4
Death, (*n*) %	(4406) 0.7	(6514) 0.8	(4860) 0.8	(7649) 0.8	(4728) 0.7	(11 545) 1.4	(39 702) 0.9

a *RGP variables are counted repeatedly, once for each of their patients. Means and percentages will thus be influenced by list size and do not describe individual RGPs.*

b *Missing data for higher education variable amount to 957 515. In addition, 5301 patients had missing data for sex and age, 5303 for country of birth, 20 341 for mean centrality, and 25 690 for all RGP variables. OOH = out-of-hours.*

**Table 3.** OR (with 95% CI) for having at least one consultation or home visit from OOH services during 2018: multilevel multiple logistic regression analysis, grouped by regular GP (RGP)

**Duration of RGP–patient relationship**	**Unadjusted**		**Adjusted for patients’ covariates^a^**		**Adjusted for patients’ and RGPs’ covariates^b^**	
	**OR**	**95% CI**	**OR**	**95% CI**	**OR**	**95% CI**
1 year (ref)						
2–3 years	0.82	0.82 to 0.83	0.87	0.86 to 0.89	0.87	0.86 to 0.88
4–5 years	0.69	0.68 to 0.70	0.80	0.79 to 0.81	0.80	0.78 to 0.81
6–10 years	0.61	0.60 to 0.62	0.77	0.76 to 0.78	0.76	0.75 to 0.77
11–15 years	0.62	0.61 to 0.62	0.78	0.77 to 0.79	0.77	0.76 to 0.78
>15 years	0.57	0.56 to 0.58	0.71	0.70 to 0.72	0.70	0.69 to 0.71

a *Adjusted for sex, age, educational level, country of birth, Charlson score, centrality, mean number of consultations per year.*

b *Adjusted for sex, age, educational level, country of birth, Charlson score, centrality, mean number of consultations per year, RGP’s sex, RGP’s age, general practice specialist, list size, vacant list capacity. CI = confidence interval. OOH = out-of-hours. OR = odds ratio.*

**Table 4.** OR (with 95% CI) for acute hospital admission during 2018: multilevel multiple logistic regression analysis, grouped by regular GP (RGP)

**Duration of RGP–patient relationship**	**Unadjusted**		**Adjusted for patients’ covariates^a^**		**Adjusted for patients’ and RGPs’ covariates^b^**	
	**OR**	**95% CI**	**OR**	**95% CI**	**OR**	**95% CI**
1 year (ref)						
2–3 years	0.91	0.89 to 0.92	0.89	0.87 to 0.90	0.88	0.86 to 0.90
4–5 years	0.89	0.87 to 0.90	0.84	0.82 to 0.86	0.83	0.81 to 0.85
6–10 years	0.92	0.90 to 0.93	0.81	0.80 to 0.83	0.80	0.79 to 0.82
11–15 years	0.93	0.91 to 0.94	0.81	0.79 to 0.82	0.79	0.77 to 0.81
>15 years	1.48	1.45 to 1.50	0.74	0.73 to 0.76	0.72	0.70 to 0.73

a *Adjusted for sex, age, educational level, country of birth, Charlson score, centrality, mean number of consultations per year.*

b *Adjusted for sex, age, educational level, country of birth, Charlson score, centrality, mean number of consultations per year, RGP’s sex, RGP’s age, general practice specialist, list size, vacant list capacity. CI = confidence interval. OR = odds ratio.*

There was a similar but somewhat weaker trend for mortality (Table 5). OR for dying decreased gradually from 0.92 (95% CI = 0.86 to 0.98) after 2–3 years’ duration to 0.75 (95% CI = 0.70 to 0.80) after an RGP–patient relationship of >15 years. Patients’ covariates were of larger influence on the results than RGPs’ covariates (Tables 3–5).

**Table 5.** Odds ratio (with 95% CI) for dying during 2018: multilevel multiple logistic regression analysis, grouped by regular GP (RGP)

**Duration of RGP–patient relationship**	**Unadjusted**		**Adjusted for patients’ covariates^a^**		**Adjusted for patients’ and RGPs’ covariates^b^**	
	**OR**	**95% CI**	**OR**	**95% CI**	**OR**	**95% CI**
1 year (ref)						
2–3 years	1.12	1.06 to 1.18	0.89	0.83 to 0.94	0.92	0.86 to 0.98
4–5 years	1.34	1.26 to 1.42	0.87	0.81 to 0.93	0.90	0.84 to 0.96
6–10 years	1.60	1.52 to 1.69	0.83	0.78 to 0.88	0.85	0.80 to 0.91
11–15 years	1.66	1.57 to 1.76	0.80	0.74 to 0.85	0.81	0.75 to 0.86
>15 years	4.03	3.81 to 4.26	0.76	0.72 to 0.81	0.75	0.70 to 0.80

a *Adjusted for sex, age, educational level, country of birth, Charlson score, centrality, mean number of consultations per year.*

b *Adjusted for sex, age, educational level, country of birth, Charlson score, centrality, mean number of consultations per year, RGP’s sex, RGP’s age, general practice specialist, list size, vacant list capacity. CI = confidence interval. OR = odds ratio.*

## DISCUSSION

### Summary

This study provides strong evidence that continuity of care by an RGP is associated with reduced need for OOH services and acute hospital admission and decreased mortality in a dose-dependent way. If the RGP–patient relationship has lasted >15 years, the probability of these occurrences is reduced by 25–30%. This effect was not significantly affected by the personal characteristics of the RGP or their list.

### Strengths and limitations

The material is large, nationwide, and more than 4.5 million individuals are included as almost all Norwegians participate in the RGP Scheme.^15^ Furthermore, the study covers a long time span with >800 000 patients having had the same RGP for >15 years. It was possible to adjust for many possible confounders by individual linking of high-quality national registries.

There is no information about the use of private health services that operate outside the national healthcare system. In some of the larger cities there are private outpatient clinics that may serve as an alternative to the public OOH services. Adjusting for the centrality variable may have reduced the importance of this factor. In Norway, private hospitals have no role in receiving patients in need of acute admissions.

Although being the most widely used measure of morbidity, the Charlson score is far from perfect. One important limitation is that psychiatric conditions are not included in the score. There is a need for a validated morbidity score based on the most important diagnoses occurring in general practice.

Nursing home residents are still formally registered with their RGP although they are cared for by the nursing home’s attending physician. This may have diluted the effect of the RGP–patient relationship for this subgroup, as nursing home residents will have been recorded with an artificially long relationship with their RGP. An opposite bias is also possible for this group: nursing home residents in need of OOH services will not be recorded by HELFO because the nursing home covers all the expenses for their residents. However, nursing home residents constitute a minor group, and this bias is likely to be of little importance.

Although the patients are listed with a named RGP of their own choice, it is still possible to see other RGPs. To what degree this may have happened over the years is not known. Visiting RGPs at other clinics rarely occurs,^12^ but if the patient’s own RGP is absent the patient may have an encounter with a colleague at the same clinic. The UPC index has been found to be 0.78 in Norway.^12^ This is higher than in Israel (0.75), Spain (0.71), England (0.61), Sweden (0.33), or Germany (0.12–0.24).^4^^,^^19^^–^22 This is to be expected since the patients have actively chosen their RGP, who on the other hand is legally obliged to provide rapid access.

The investigated associations are complex, and therefore many possible confounders have been adjusted for. Patients’ age and morbidity are examples of such confounding variables that may determine use of OOH services, hospital admission, and death. In addition, such variables may have a bearing on the duration of the RGP–patient relationship and thus obscure its effect on the outcome. Sex, ethnicity, and education level are other variables that may influence health, the use of health services, and mortality. Since there are geographical differences in the use of hospitals and OOH services,^23^^,^^24^ the analysis also adjusted for a rural–urban variable (centrality). It is possible that healthy patients with few visits may have long continuity without the benefit of being well known by their RGP. Therefore, the frequency of visits to the RGP has been included as a patient-related adjustment variable.

It is also possible that doctor-related factors may influence the outcome. Therefore, the RGPs’ sex, age, and general practice specialisation were included as adjustment variables. The number of patients listed with the RGP may affect accessibility, and the number of vacant list places may be taken as an indicator of the RGP’s popularity and competence. Both these variables were therefore included as adjustment variables.

### Comparison with existing literature

In a survey of 133 Norwegian GPs, Hjortdahl^25^ found that it took at least 1 year, and often 5, to create an extensive knowledge base about individual patients. In parallel, he found that the duration of the patient–doctor relationship was associated with patient satisfaction, which also could take as much as 5 years to develop.^26^ The present study indicates that even much longer relationships may be of additional benefit.

Many previous studies have found an association between continuity of care and lower use of OOH services, use of emergency departments, and acute admissions. Most of these studies cover a limited time span, and continuity is usually defined by the UPC index or similar measures.^4^^–^6^,^^22^^,^^27^^–^30 The authors are not aware of any studies comparable with this one, investigating the time span that patients have been formally listed with a named physician.

In the UK, patients are usually listed with a general practice, not a named RGP. However, in 2014 an RGP scheme for patients aged ≥75 years was tried in 139 practices and evaluated after 2 years.^31^ The end result was that personal continuity did not improve and there was no decrease in acute hospital admissions.

In a survey of 8068 older Americans, it was found that 55.3% had a tie to their physician of >5 years, and 35.8% ≥10 years.^32^ Longer duration was associated with lower cost and lower risk of hospital admission, but not for use of emergency room. Multivariate analyses, adjusted for sociodemographic and clinical covariates, failed to demonstrate a dose–response relationship between duration of tie and any of the outcomes measured.

Pereira Gray *et al*^3^ reviewed several studies that reported significantly lower mortality with increasing continuity of care. Most of these studies used the UPC index as a measure of continuity. A possible important confounding factor in such studies is increased number of contacts with a specific physician because of increased morbidity before death, described as reverse causality bias. This source of bias is avoided by the longitudinal design of the present study, not counting number of contacts. In addition, morbidity was recorded during the 3 preceding years (2015–2017).

One long-term Dutch study followed older adults for up to 17 years and found increased mortality among those with low continuity of care.^33^ However, that study included only 1712 subjects, and the association was barely statistically significant. Earlier studies of large populations have used aggregated data at practice or primary care trust level. In such studies, lower mortality has been found for patients recalling access to their preferred GP.^34^^,^^35^

### Implications for research and practice

When patients can choose their personal RGP, the ground is laid for a lasting relationship. Over the years, RGPs may become specialists on their individual patients, rather than on their diseases. This is the essence of personal doctoring, also described by William Osler: *‘It is much more important to know what sort of a patient has a disease than what sort of a disease a patient has.’*^36^ The World Health Report 2008 also emphasised a stable, long-term, personal relationship as a prerequisite for providing patient-centred care.^37^

Longitudinal continuity is not possible without physicians who remain in their practices over time. The promotion of stability among RGPs should be a priority for health authorities. A recent study from Norway demonstrated that even short breaks in continuity entailed increases in use of OOH services and admissions for ambulatory care-sensitive conditions.^38^ More research is needed on what measures may be undertaken to promote stability of RGPs.

The Norwegian RGP Scheme was inspired by similar systems in countries such as the UK, Denmark, and the Netherlands. Although there may be some differences, the results of the present study are probably representative for those who have organised primary care in this way. Other countries may be inspired to develop and strengthen general practice in their healthcare systems. Interpersonal continuity of care should be encouraged.
